# A Novel Mouse Segmentation Method Based on Dynamic Contrast Enhanced Micro-CT Images

**DOI:** 10.1371/journal.pone.0169424

**Published:** 2017-01-06

**Authors:** Dongmei Yan, Zhihong Zhang, Qingming Luo, Xiaoquan Yang

**Affiliations:** 1 Britton Chance Center for Biomedical Photonics, Wuhan National Laboratory for Optoelectronics-Huazhong University of Science and Technology, Wuhan, Hubei, China; 2 Key Laboratory of Biomedical Photonics of Ministry of Education, Huazhong University of Science and Technology, Wuhan, Hubei, China; University of Chicago, UNITED STATES

## Abstract

With the development of hybrid imaging scanners, micro-CT is widely used in locating abnormalities, studying drug metabolism, and providing structural priors to aid image reconstruction in functional imaging. Due to the low contrast of soft tissues, segmentation of soft tissue organs from mouse micro-CT images is a challenging problem. In this paper, we propose a mouse segmentation scheme based on dynamic contrast enhanced micro-CT images. With a homemade fast scanning micro-CT scanner, dynamic contrast enhanced images were acquired before and after injection of non-ionic iodinated contrast agents (iohexol). Then the feature vector of each voxel was extracted from the signal intensities at different time points. Based on these features, the heart, liver, spleen, lung, and kidney could be classified into different categories and extracted from separate categories by morphological processing. The bone structure was segmented using a thresholding method. Our method was validated on seven BALB/c mice using two different classifiers: a support vector machine classifier with a radial basis function kernel and a random forest classifier. The results were compared to manual segmentation, and the performance was assessed using the Dice similarity coefficient, false positive ratio, and false negative ratio. The results showed high accuracy with the Dice similarity coefficient ranging from 0.709 ± 0.078 for the spleen to 0.929 ± 0.006 for the kidney.

## Introduction

Multi-modality imaging techniques have been widely used in preclinical research owing to their advantage in providing complementary information. As a predominant structural imaging modality, micro-CT has often been combined with various functional imaging modalities including positron emission tomography (PET) [1], single photon emission computed tomography (SPECT) [2], and optical imaging such as fluorescence molecular tomography (FMT) [3] and bioluminescence tomography (BLT) [4]. In a hybrid imaging system combining FMT and micro-CT, the anatomical information provided by micro-CT can be used to locate the fluorophore distribution as a reference [3], construct a more accurate solution to the forward problem [5, 6], and reduce the ill-posedness of the inverse problem by locating borders where the optical characteristics might change [6, 7]. Thus, it is necessary to obtain the locations and boundaries of internal organs in mouse micro-CT images.

Unfortunately, the numerous segmentation methods proposed for clinical CT images [8–10] can hardly be applied in micro-CT, in which the contrast-to-noise ratio is significantly reduced by the low radiation dose delivered to the small animals [11]. Since the low soft tissue contrast limits the segmentation of mouse internal organs from micro-CT images, many studies have used atlas-based registration methods to help estimating the internal organs instead of accurate segmentation. Most of these methods use high-contrast organs to register the atlas to individual subjects. The low-contrast organs are subsequently estimated. A method that took special care of the skeleton was presented in Li et al. [12]. They non-rigidly registered centerline representations of skeletons and used the correspondences to define a Thin-Plate-Spline (TPS) mapping. Baiker et al. [13] used skin, skeleton, and lung together as aligning features. To overcome large variations in posture, they developed an articulated skeleton atlas. The individual bones as well as lung were registered one by one. The skin was initialized constrained by the skeleton. Then a TPS based transformation was obtained and used for atlas mapping. Similarly, a 3-D shape context-based nonrigid registration method was proposed to register skeleton, lung, and skin, and a thin-plate-spline (TPS) transformation was built to map all organs [14]. Wang et al. [15] constructed a statistical atlas based on multiple training subjects to include inter-subject anatomical variations. Thus, the proposed statistical shape model and conditional Gaussian model-based image registration method yielded higher accuracy. They also presented a fully articulated atlas based on multiple training subjects [16].

Besides atlas-based registration, several authors perform accurate segmentation for analyzing organs quantitatively in normal and diseased model, providing a heterogeneous tissue model for optical tomography reconstruction, and so on. Freyer et al. [17] identified bone structures, lung, and heart with a conventional thresholding method, seed growing algorithm, and a static heart model, respectively. To segment more low-contrast soft tissues, researchers used iodinated lipid emulsion contrast agents [18] or nanoparticulate contrast agents [19] to enhance the contrast. Thus, organs can be segmented accurately with conventional approaches. These agents are always expensive and unavailable. The low clearance rate of this type of agent limits its applications in hybrid imaging systems because the residual contrast agents may influence the functional imaging. In addition, if the functional imaging is performed before administration or after the clearance of contrast agents, the dual-modal imaging need to be implemented separately. Thus, the structural image provided by the enhanced micro-CT cannot be merged directly with the functional image, and image registration is needed.

To overcome these limitations, we advocate a new segmentation scheme based on different dynamic enhancement characteristics between organs after injection of a non-ionic iodinated contrast agent. The non-ionic iodinated contrast agents have advantages of low cost, low toxicity, easy availability, and high clearance rate. They have been widely used in preclinical studies such as vascular imaging [20–22] and tumor angiogenesis [23, 24]. The technique of dynamic contrast enhancement have been used in computer-aided diagnosis of breast lesions [25] and tumor segmentation in magnetic resonance imaging (MRI) [26, 27]. This technique used for mouse micro-CT image segmentation would provide a practical solution to extract structural priors from micro-CT images for optical tomography reconstruction in the dual-modality system.

**Fig 1:**
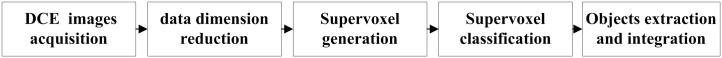
Flow chart of the image segmentation scheme. The proposed segmentation algorithm includes five consecutive steps: DCE micro-CT images acquisition, data dimension reduction, supervoxel generation, supervoxel classification, and target organs’ extraction.

The segmentation scheme based on the dynamic contrast enhancement method is shown in Fig 1. Iohexol, a non-ionic iodinated contrast agent, is injected via a tail vein of the mouse. A homemade micro-CT is used to acquire the dynamic contrast enhanced (DCE) images. Then, a principal component analysis (PCA) is performed to reduce data dimensions and noise. Supervoxel generation is implemented to provide primitives for classification. Subsequently, supervised learning algorithms are used to classify the supervoxels into different categories according to the supervoxels’ signal intensities at different time points. The heart, liver, spleen, lung, and kidney are extracted separately from the corresponding categories through morphological processing. The bone is segmented by a thresholding method. Finally, all organs were integrated, forming an intact mouse anatomy. The method was validated on seven BALB/c mice and two different classifiers.

## Materials and Methods

### Ethics statement

All of the experiments were conducted in accordance with the Institutional Animal Care and Use Committee of Hubei Province. The protocol was approved by the Institutional Animal Care and Use Committee at Tongji Medical College, Huazhong University of Science and Technology (Permit Number: 452). All efforts were made to minimize suffering during the study procedure.

### Image acquisition

We used a homemade gantry rotation micro-CT system to acquire the DCE images. The system consists of a micro-focus X-ray tube (Apogee 93501, Oxford Instrument, U.S.) and a complementary metal-oxide-semiconductor (CMOS) based flat-panel detector (Dexela 1512, PerkinElmer, U.K.) that are fixed on a rotation stage (ADRT-260-180, Aerotech, U.S.). The mouse position is adjustable using a three-dimensional translational platform. Source-to-detector distance and source-to-object distance are 632.1 mm and 332.7 mm, respectively, contributing to a magnification factor of 1.9. The field of view is 60.5 mm in diameter and 76.5 mm in length.

The heart rate of mice is several times greater than that of humans. Consequently, the non-ionic iodinated contrast agents could be cleared from the vessels of mice in several minutes. In order to acquire DCE images, the scanner was operated using the following high temporal resolution settings: X-ray tube settings of 50 kVp and 800 μA with 1 mm aluminum filter, a 40 ms exposure time per projection, 400 projections, a 360° continuous rotation, a binning factor of 4, and no gating. Without a sliding ring, each scan ultimately took about 45 s. All images were reconstructed using the Feldkamp’s filtered back-projection algorithm accelerated by a GPU [28], resulting in a matrix size of 200×200×450 with an isotropic voxel size of 157.5 μm.

Seven female BALB/c mice with body weights ranging from 14 to 18 g were used for demonstration of the proposed method. A dose of 1 g/kg urethane and 0.2 g/kg α-chloralose was administered intraperitoneally to anesthetize the mouse. Then, a polyethylene catheter, connected with a needle at one end and the tube of a 1 ml syringe at the other end, was inserted into the lateral tail vein and fixed with tape for contrast agent delivery. Each mouse lay prone on the sample holder. The tail with the needle and catheter was kept outside the scanner’s field of view. Dynamic image sequence was acquired using the following protocol: after acquisition of the pre-contrast series, iohexol (300 mg I/ml, GE, Shanghai, China) was administrated by a syringe pump (KDS 210, KD scientific, Holliston, MA, USA) at a dose of 25 μl/g and a constant injection duration of 45 s, triggered at the start of the first post-contrast scan. A total of five post-contrast series were obtained with an interval of 50 s. Following the scanning process, mice were revived on a heating pad and returned to their home cages.

During the image acquisition process, some motion, due to breathing and cardiac motions, is likely to occur. Although algorithms [29, 30] exist to correct the motion, no registration algorithms were used in this study.

### Principal component analysis

As a simple and nonparametric method, principal component analysis (PCA) can be used to reduce the dimensionality of large multivariate datasets and retain the variability at the same time through constructing new uncorrelated variables, namely principal components (PCs) [31]. For each mouse, we reshaped the 3-D image matrix at one time point to a column vector and organized the six column vectors from six time points into a two-dimensional matrix, **I**(*X*, *t*), with the individual signal versus time curves in its rows (*X* depicting spatial location and *t* depicting time). PCA decomposes **I** into the product of two parts: one of the parts consists of the PCs, which are orthonormal and ordered by decreasing amounts of the variability they represent; the other part is named scores and consists of the weights of each PC in the original data.

We chose the first four PCs, which explained more than 99% of total variability in **I**, resulting in a new dataset consisting of four scores for each voxel. Thus, the feature dimension reduced from six to four. Then, for each PC, the corresponding scores for the volume were normalized between [0, 1] and reshaped reversely to a 3-D image matrix for subsequent supervoxel generation. The advantage of the PCA here is its ability to remove the effects of noise and also to accelerate the following supervoxel generation and classification by reducing feature dimensions.

### Supervoxel generation

Superpixel/supervoxel algorithms provide a convenient primitive to increase the granularity and overcome the influence of noise effectively. Furthermore, superpixels capture the local redundancy and reduce the computational complexity for further processing. The simple linear iterative clustering algorithm (SLIC) [32] was used in this research. The SLIC algorithm adapts a *k*-means clustering approach to generate 3-D supervoxels. This algorithm has been shown to be a fast method with good performance. SLIC initializes *k* cluster centers by sampling the grid regularly with distance $S=\sqrt[{3}]{N/k}$ in all three dimensions, where *N* denotes the number of voxels. Next, the centers are moved to the lowest gradient position in a 3×3×3 neighborhood, followed by iterative clustering. A distance function is defined, combining the spatial and intensity proximity of voxels within a limited 2*S*×2*S*×2*S* region. Ultimately, post processing is applied to enforce connectivity.

The SLIC was extended to an n-feature image to enable extraction of supervoxels from DCE micro-CT images [26]. A distance function combining feature distance and spatial distance was defined as follows:

$$D=\sqrt{\frac{\frac{1}{n}\sum_{k=1}^{n}{\left(b_{jk}-b_{ik}\right)}^{2}}{m}+\frac{\sum_{k=1}^{3}{\left(x_{jk}-x_{ik}\right)}^{2}}{S}}$$ (1)

where *b*_*jk*_ is the score of the j*th* voxel corresponding to the k*th* PC, (*x*_*j1*_, *x*_*j2*_, *x*_*j3*_) is the 3-D coordinate of the j*th* voxel, and *m* is a constant allowing us to weigh the relative importance between color similarity and spatial proximity. Normalizing the spatial proximity and feature similarity by *S* and *m* allows the distance measurement to combine these quantities, which have very different ranges. *S* and *m* provide control over the size and compactness of the supervoxels, respectively.

As presented above, we used n = 4 components, and the scores were between [0, 1]. In our experiments, *S* was chosen to be 8 by qualitatively assessing the ability of the supervoxel algorithm to correctly separate thin structures, and *m* was chosen to be 0.02 empirically to provide a good boundary adherence.

### Supervoxel classification

After the supervoxels were generated, they were classified into different categories according to the signal intensities at different time points, which were represented by the scores of the first four PCs as previously mentioned. The mean value of all voxels in each supervoxel were calculated and used as the feature vectors. Based on the characteristics of the acquired DCE images, we planned to classify the image into ten categories, which were expected to correspond to heart, liver, spleen, lung, kidney, bone, intestinal wall, intestinal cavity, subcutaneous fat and muscle, and some small regions that adhere to skin that showed irregular changes because of physiologic motion. The training sets were recognized and selected manually from the images to be segmented. In consideration of the organ heterogeneity, for each category, the training set must contain supervoxels from different parts. For example, some supervoxels from the renal parenchyma and renal pelvis were both selected and labeled as kidney in the training set. In order to evaluate the influence of the training sample size, classifications using different numbers of training samples were compared.

The classification was performed with two different classifiers: a support vector machine (SVM) classifier with radial basis function (RBF) kernel and a random forest (RF) classifier.

SVM classifier with RBF kernel: SVM has strong generalization ability and presents advantages in solving small sample, nonlinear, and high dimension pattern recognition problems. An open source library LIBSVM [33] was used in our research. The cost parameter, *C*, and the shape parameter, *γ*, of the RBF kernel function were found using a grid search. Twenty-one different *C*-values, ranging from 2^−5^ to 2^15^, and 31 different *γ*-values, ranging from 2^−15^ to 2^15^, were tested, using a 5-fold cross-validation. The (*C*, *γ*)-pair that gave the smallest misclassification fraction was chosen for the SVM model. The probability estimates were generated from the SVM decision function.

Random forest (RF) classifier: The RF classifier was included since it incorporates feature selection as a part of its learning and classification algorithms [34]. RF classifiers do not overfit because of the law of large numbers. A RF is an ensemble of decision trees. The number of features selected at each node is recommended to be much less than the total number of features. Therefore, we set it to 2 to ensure the ability of a single decision tree. In addition, we tested the number of trees by 5-fold cross-validation and found 500 trees were enough to ensure the accuracy. The likelihood given by the classifier is computed by averaging the output over all the trees.

### Objects extraction and integration

The value for each supervoxel was used as the values for all voxels in the supervoxel. For both classifiers, instead of directly using the classification results, the probabilistic outputs were used. A Gaussian filter was applied on the probability maps, and the voxels were finally labeled to the class with maximum probability. Given that the initial interval, *S*, for supervoxel generation was set to be 8 voxels, we used a 9×9×9 Gaussian filter with σ = 3.

The final step of our approach is to extract the organs from the corresponding classes by a simple post-processing procedure and then integrate them. For the heart, liver, spleen, lung, and kidney, the procedure was the same. First, a binary image was obtained from the classification results. Then, an object opening using a disk-shaped structure element with a radius of 2 voxels was implemented. Finally, an 8-neighborhood connected component analysis was applied, and holes in the objects were filled.

The bone has many tiny structures. Therefore, it cannot be obtained exactly using a similar procedure. Fortunately, the bone segmentation can be performed on images by a simple thresholding method. Due to the physiological motion, the bone might have small position displacements between different images. For the pre-contrast image, the threshold could be simply determined by the histogram. For the post-contrast images, to avoid the influence of other enhanced organs, the region of interest should be determined in advance by dilating the segmented bone in the pre-contrast image. Then, the threshold segmentation could be implemented in this region.

The subcutaneous fat and muscle and the intestine could not be classified and extracted successfully in this research due to their less prominent enhancement characteristics and anatomical characteristics. After all target organs had been extracted separately, they were integrated into a whole mouse volume of data, and overlaps were eliminated. The priority order was bone, kidney, lung, liver, heart, and spleen, and this order was decided according to the segmentation accuracy and stability.

### Validation of the image segmentation

To evaluate our algorithm, we compared the results to the reference datasets segmented manually. In order to quantify the intra-operator variability of manual segmentation, two manual segmentation repetitions were carried out by one expert. For inter-operator variability, a comparison was performed between the segmentation results of two experts. Therefore, a total of three manual segmentations were achieved, which we called M1, M2, and M3, respectively. M1 and M2 were from the first expert, and M3 was from the second expert.

The manual segmentation was performed mainly based on the post-contrast image at 100 s on a 3-D visualization and analysis software platform (Amira, version 5.2.2, Germany). Three metrics, Dice similarity coefficient (DSC), false positive ratio (FPR), and false negative ratio (FNR), were used for accuracy assessment. They are defined as follows:

$$\text{DSC}:\;\frac{2\left|X\cap Y\right|}{\left|X\right|+\left|Y\right|}$$ (2)

$$\text{FPR}:\;\frac{\left|X\cup Y\right|-\left|Y\right|}{\left|Y\right|}$$ (3)

$$\text{FNR}:\;\frac{\left|X\cup Y\right|-\left|X\right|}{\left|Y\right|}$$ (4)

where *X* and *Y* represent the segmented dataset and the reference dataset, respectively, and |•| denotes the number of voxels.

DSC measures the similarity between the two datasets and is a more general measure for accuracy. FPR indicates the percentage of voxels of the segmented data that do not belong to reference data, while FNR indicates the percentage of voxels of the reference data that are not contained by segmented data.

## Results

### Image acquisition

**Fig 2:**
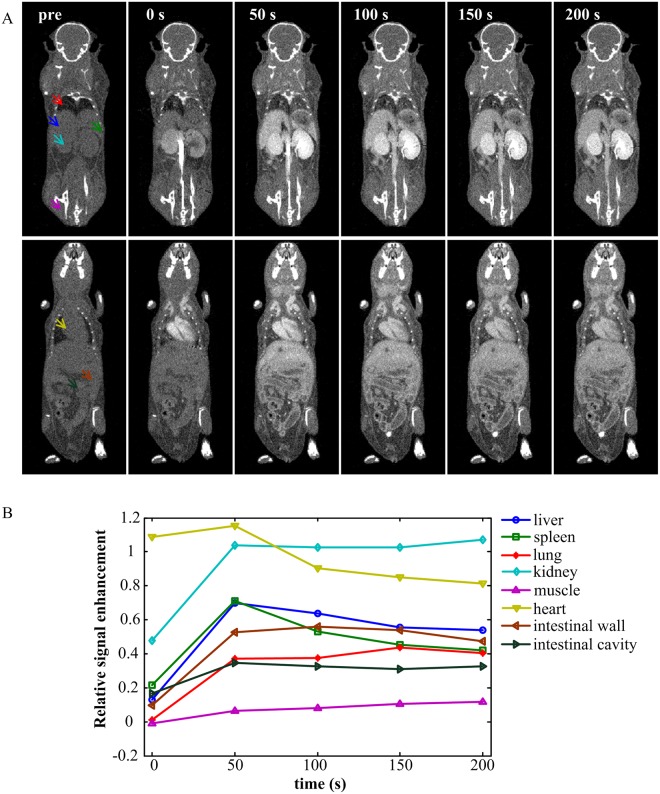
Dynamic contrast enhancement procedure after contrast agent administration. (A) Representative coronal micro-CT images before contrast agent injection and at 0 s, 50 s, 100 s, 150 s, and 200 s post-contrast injection. The image at 0 s was acquired during the inflow of contrast agent. All of the images are displayed with the same gray scale window. (B) The relative signal enhancement versus time curves of regions depicted by the arrows in (A) with the same colors.

The images before and after contrast agent administration are shown in Fig 2A. The image at 0 s represented the first post-contrast image acquired during the inflow of contrast agent. Fig 2B represents the relative signal enhancement versus time curves of the regions whose centers were marked by arrows with same color as in Fig 2A. The relative signal enhancement at a specific time point *t* is defined as follows:

$$R_{t}=\frac{T_{t}-T_{0}}{T_{0}}$$ (5)

where *T*_*0*_ and *T*_*t*_ represent the intensity of pre-contrast and post-contrast at time point *t*, respectively. To avoid the influence of noise, we used the mean intensity of a region containing 100 voxels to calculate *R*_*t*_. The curves demonstrate that the heart is enhanced immediately after injection, and the intensity of the kidney remains high for a longer time. The intensity of the spleen has a steeper drop in intensity than the liver. The subcutaneous muscle and the intestinal cavity have little enhancement.

In our experiments, all mice tolerated the injection of 25 μl/g of iohexol, and no immediate behavioral changes were observed. As to the radiation dose, the exposure time was so short that the radiation dose was still very low even though we scanned six times for each mouse. We measured the radiation dose in a mouse carcass with a thermoluminescence dosimeter (CTLD-1000). The detector was calibrated at the National Institute of Metrology of P. R. China. The total dose of six scans is 53.52 mGy, which is only about 1% of the LD50/30 for a small rodent [11].

### Supervoxel generation

**Fig 3:**
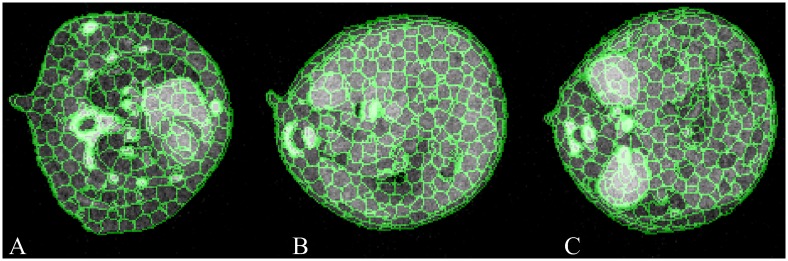
Results of supervoxel generation shown in 2-D axial slices. The green denotes the boundaries of supervoxels. (A) Axial slice of thorax containing the heart and lung. (B) Axial slice of upper abdomen containing the liver and spleen. (C) Axial slice of lower abdomen containing the kidney and intestine.

Fig 3 shows the supervoxel generated by the distance function and parameters we chose in this research. The green curves denote the boundaries of supervoxels, and the three panels represent three axial slices containing different anatomic structures. It could be seen that the supervoxels adhere well to the organ boundaries. Note that supervoxels are 3-D, yet the figure shows three different 2-D slices of supervoxels.

### Influence of the number of training samples

**Fig 4:**
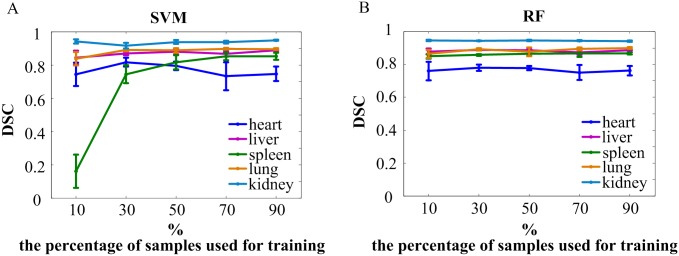
Influence of the number of training samples on segmentation accuracy. 10%, 30%, 50%, 70%, and 90% of the total samples of each category were selected randomly from the total data set and consisted of training sets for classification. Each case was repeated five times. The means and standard deviations of Dice similarity coefficients for the heart, liver, spleen, lung, and kidney were calculated (compared with M1). (A) The DSC of the organs classified by SVM. (B) The DSC of the organs classified by RF. For the lung, one value at 10% and two values at 30% were excluded from statistics because it failed to extract the lung by post-processing.

We used a different number of training samples for classification and evaluated the influence of the number of training samples on the accuracy of our segmentation method. The test to evaluate the influence of the number of training samples was performed on one mouse. First, for each of the ten categories, a relatively large number of supervoxels (Table 1) were recognized from the enhanced images and chosen manually to constitute the total data set. Next, five subsets that contained 10%, 30%, 50%, 70%, and 90% of the total number of each category were used as training sets. For each subset, the samples were selected randomly and repeated five times. Finally, these training sets were used for classification, and the target organs were extracted by post-processing. Table 1 lists the number of supervoxels for each category in the total data set. The means and standard deviations of the Dice similarity coefficients compared with M1 for each training number are plotted in Fig 4 and listed in S1 Table. Apparently, the SVM results in a low accuracy for the spleen when the training sample size is too small. In addition, the RF could not extract the lung successfully due to its connection with subcutaneous tissue being misclassified. This happened once when 10% of samples were chosen and twice when 30% of samples were chosen. The corresponding DSC values were excluded. In other cases, the number of training samples has little influence on the final results.

**Table 1:** Number of supervoxels for each category chosen to constitute the total data set.

**Category**	Heart	Spleen	Kidney	Intestinal wall	Subcutaneous fat and muscle
**Number**	36	19	36	60	76
**Category**	Liver	Lung	Bone	Intestinal cavity	Small regions adhering to skin
**Number**	54	40	34	60	36

### Visual assessment of segmentation results

**Fig 5:**
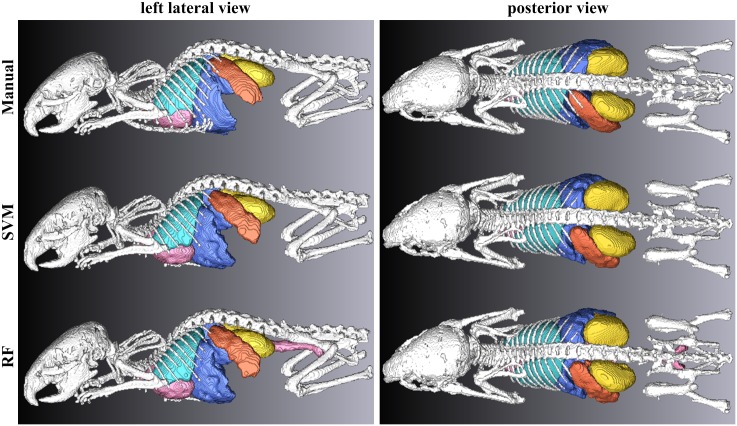
Visual comparison of the segmentation results with the reference datasets, shown in 3-D isosurface rendering. Left column: left lateral view. Right column: posterior view. Top row: manual segmentation (M1). Middle row: segmentation obtained by the SVM. Bottom row: segmentation obtained by the RF.

**Fig 6:**
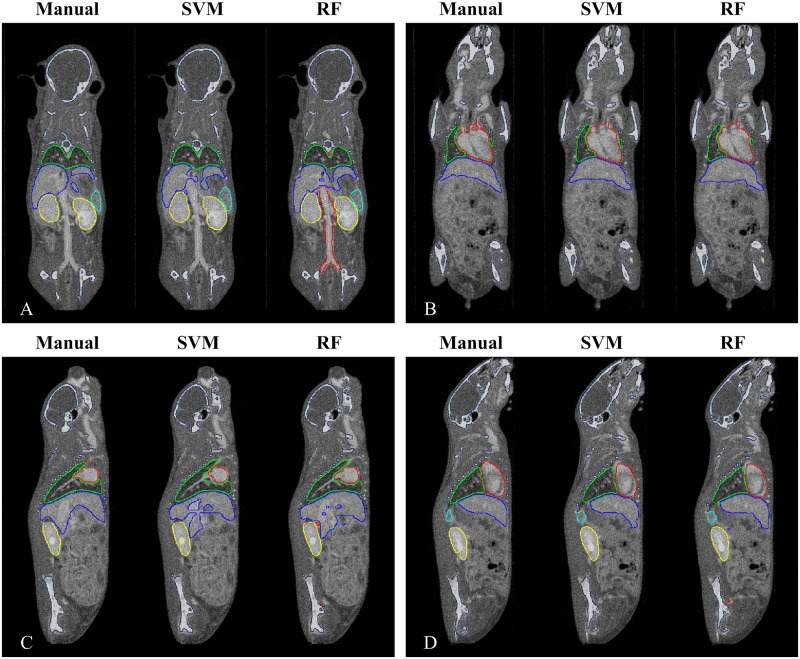
Visual comparison of the segmentation results with the reference datasets, shown in 2-D images. The organ boundaries of manual segmentation (M1) and automatic segmentation based on the SVM and the RF are superimposed on two coronal images (A, B) and two sagittal images (C, D).

Fig 5 and S1–S3 Movies show a 3-D isosurface rendering of organs obtained by manual segmentation (M1) and our automatic segmentation using the SVM and RF. Fig 6 shows the segmented organ boundaries superimposed on contrast-enhanced images. Coronal and sagittal slices are presented. The segmentation of the lung and kidney were highly consistent with the ground truth. The heart and liver seemed to be influenced by large blood vessels, such as the posterior vena cava. In addition, the spleen had a larger region than the ground truth.

### Quantitative assessment of segmentation results

**Fig 7:**
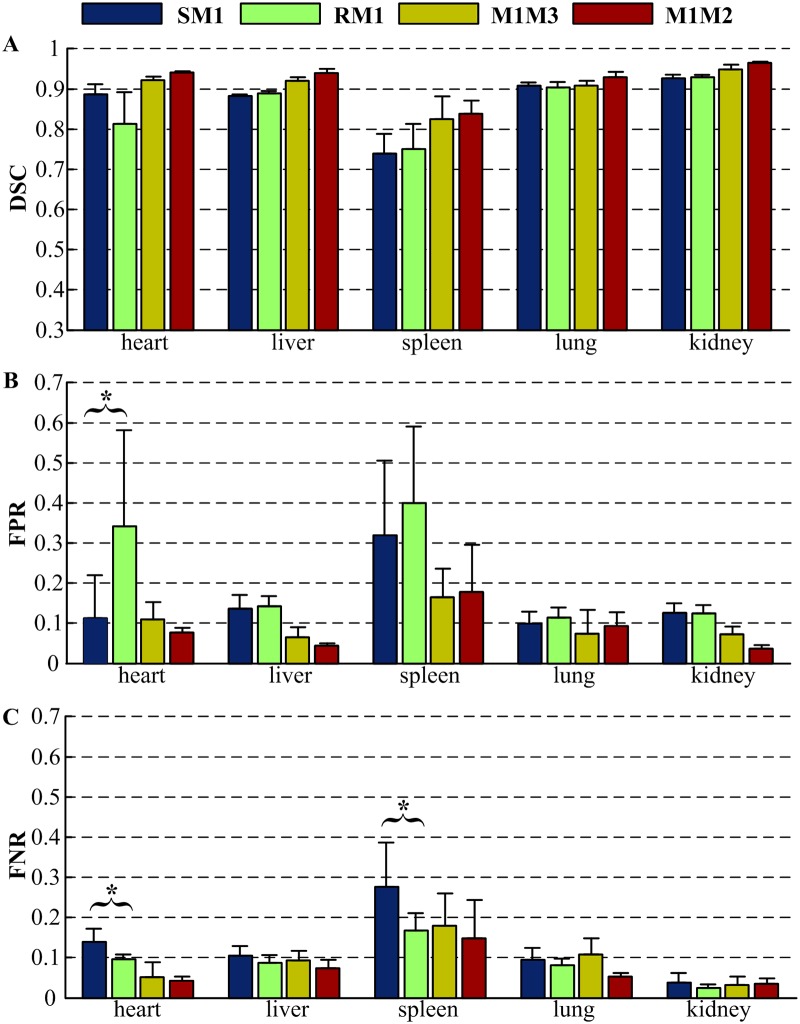
Quantitative evaluation of the proposed methods by comparison to manual segmentation. ‘SM1’ and ‘RM1’ represents the comparison of the automatic segmentation by SVM and RF with the manual segmentation (M1), respectively. ‘M1M3’ compares the manual segmentations of two independent experts. ‘M1M2’ compares two manual segmentation repetitions of one expert. (A) Dice similarity coefficient. (B) False positive ratio. (C) False negative ratio. (* Indicates *p* < 0.05.)

According to the evaluation results of the influence of the training sample size, the training sample size used for all seven mice in our research was equivalent to about 50% of the total number. The quantitative evaluation of our method based on the two different classifiers is listed in S2 Table and illustrated in Fig 7. The mean values and standard deviations of DSC, FPR, and FNR are plotted. ‘SM1’ and ‘RM1’ represents the comparison of the automatic segmentation by SVM and RF with the manual segmentation (M1), respectively. ‘M1M3’ quantifies the inter-operator variability by comparing the manual segmentations of two independent experts. M3 was taken as reference dataset to compute the FPR and FNR. Similarly, ‘M1M2’ quantifies the intra-operator variability by comparing two manual segmentation repetitions of one expert, taking the M2 as reference dataset. For the DSC, a mean value closer to 1 means better accuracy. However, for the FPR and FNR, a mean value closer to 0 means better accuracy. The two classifiers yield similar DSC values that are above 0.700 for all organs, which means our results and the ground truth are in good correspondence [35]. The highest accuracy is obtained for the kidney with a DSC of 0.929, a slightly lower than the DSC between two operators (0.949). Moreover, for the lung, the variability between the automatic and manual segmentation (0.904) is comparable to the inter-operator variability (0.908). Worse result is obtained for the spleen with a low DSC value of 0.709. For the bone, the manual segmentation was only performed in M1. Compared to M1, the bone segmented by the thresholding method yielded a large DSC of 0.925 ± 0.013. The FPR and FNR were 0.083 ± 0.041 and 0.068 ± 0.032, respectively.

In order to evaluate the significance of difference between the SVM and RF, a Wilcoxon rank sum test for ‘SM1’ *vs* ‘RM1’ was performed with each organ for each of the three accuracy values. Most of the tests yielded results of *p* > 0.05, with only three exceptions: the results of the heart FPR and FNR were *p* = 0.0233 and *p* = 0.0122, respectively, and the result of the spleen FNR was *p* = 0.0262.

## Discussion and Conclusions

In this manuscript, we proposed a robust automatic segmentation framework for mice based on DCE micro-CT images. Firstly, DCE images were acquired by a homemade micro-CT system. Then, PCA was used to reduce data dimensions, and the image was over-segmented to supervoxels. Subsequently, supervoxels were classified into different categories based on signal enhancement characteristics. Finally, the heart, liver, spleen, lung, and kidney were achieved by simple morphological post- processing, and the bone was segmented by a thresholding method. Seven mice were used to validate the method.

Fig 4 illustrates the influence of training set size on the accuracy of segmentation. It is clear that the spleen yields a very small DSC value by the SVM when the training set contains too few samples. In addition, we found that the lung could not be extracted successfully by morphological processing when the RF was applied with a small training set size. This result provides a guidance for decision in the training set size.

The quantitative evaluation demonstrates that our method has high accuracy. Judging from the results of the Wilcoxon rank sum test for ‘SM1’ *vs* ‘RM1’, the two classifiers, SVM with RBF kernel and RF, yield similar DSC without significant difference. As shown in Fig 7, the liver, lung, and kidney achieved remarkable high DSC values (>0.850) and low FPR and FNR values (<0.150), which indicates excellent agreement between the automatic segmentation and manual segmentation, only a little worse than the agreements between two independent manual segmentation. The heart yielded a suboptimal DSC value and a high FPR when it was segmented based on the RF. The segmentation errors occurred mostly due to the influence of large vessels close to the heart, which could be reduced by adjusting the parameter of post-processing. For example, the radius of structure element used for object opening can be increased to separate the vessels from the heart. The poor performance of the spleen segmentation was induced by its similar intensity change with surrounding tissues, such as pancreas. In addition, due to its small size, small disagreement would lead a great decrease of the DSC value, which could be proved by the large inter- and intra-operator variability of the spleen manual segmentation. The bone segmented by a thresholding method produced a large DSC value of 0.925 ± 0.013.

To date, many efforts have been made to improve the accuracy of atlas-based registrations [12–16]. The performance of registration methods is highly dependent on the relationship between the atlas and the animals. Different strains of the animals need a different atlas. To mitigate individual differences of animals from the same strain, a large number of the animal model must be included, and the posture of the animals must be the same. Baiker et al. [13] had developed an articulated skeleton atlas to overcome large variations in posture. However, the result still had poor accuracy. The DSC between manually segmented and interpolated organs varied between 0.470 ± 0.080 for the kidneys and 0.730 ± 0.040 for the brain, which is far lower than our accuracy. A statistical atlas based on 45 training subjects was constructed by Wang et al. [15] and used to yield high accuracy registration. The lung and heart had a DSC as high as 0.900. However, for the abdominal organs, the DSC was below 0.800. The spleen only had a DSC of 0.450. In contrast, our method has the advantages of simple algorithms and high accuracies without restriction of variety for individual differences and postures of the animals. Except for atlas-based registrations, a few studies on accurate segmentation of whole mice CT images are found in literatures [17–19]. The small-animal-dedicated contrast agents, such as iodinated lipid emulsion contrast agents [18] and nanoparticulate contrast agents [19], are often expensive and unavailable. Moreover, due to the low clearance rate, this kind of agent is not suitable for daily repeated imaging and multi-modality imaging. Many simulations [6, 18, 36], phantom experiments [7, 37], and *ex vivo* experiments [6, 38] have proved that the anatomical structures extracted from micro-CT provided *a priori* for the FMT to reconstruct images with accurate localization and quantification of fluorophore distribution. Owing to the high clearance rate of the non-ionic iodinated contrast agents, the DCE micro-CT images can be acquired immediately after the FMT imaging without moving the mice. Thus, the anatomical information and functional information can be merged directly according to the dual-modal system geometry. As a result, the proposed method could further promote *in vivo* experiment researches in dual-modal micro-CT/FMT imaging.

It is worth noting that our method still has some limitations. First, no registration for the 4-D images is implemented to correct the breathing and cardiac motion. Second, our method relies on the temporal resolution of the micro-CT scanner due to the rapid clearance rate of the contrast agent. From the curves in Fig 2, we could conclude that a scanner with one-minute temporal resolution is able to capture enough information of different organs in our research for classification. In addition, the administered volume of contrast agent in our research is relatively large, almost 30% of the LD50 [39]. Although all the mice in our experiments tolerated this dose well and no immediate behavioral changes were observed, the adverse effects were not further investigated. However, it can be expected that these limitations will be overcome along with the fast development of the CT techniques. So far, some commercially available scanners are fast enough and have been used in vascular imaging enhanced by non-ionic iodinated contrast agents [21, 40]. High performance reconstruction methods, which need fewer exposures, will also reduce the requirements of imaging speed and contrast agents [41, 42].

## Supporting Information

S1 Movie 3-D isosurface rendering of organ volumes segmented manually. To allow clear visualization, the bone has been removed.
(MPG)

S2 Movie 3-D isosurface rendering of organ volumes segmented by SVM. To allow clear visualization, the bone has been removed.
(MPG)

S3 Movie 3-D isosurface rendering of organ volumes segmented by RF. To allow clear visualization, the bone has been removed.
(MPG)

S1 Table DSC values of different organs segmented by SVM and RF with different training set size (compared to M1). (XLS)

S2 Table Three accuracy values, DSC, FPR, and FNR for ‘SM1’, ‘RM1’, ‘M1M2’, and ‘M1M3’ of all seven mice. (XLS)

S3 Table The *p* values of the Wilcoxon rank sum test for ‘SM1’ *vs* ‘RM1’ performed with each organ for each of the three accuracy values. (XLS)
